# Sleep characteristics in young children with autism spectrum disorder: a case control study

**DOI:** 10.3389/fped.2026.1742989

**Published:** 2026-09-02

**Authors:** Xian Liu, Cheng Guo, Cuilian Gao, Yunting Li, Fei Ren, Shenjie Yu, Qinghuan Ding, Yuenuo He, Zimo Li, Zhifang Huang, Jing Yu, Wenqing Wei, Jingyu Huang, Wen-Xiong Chen

**Affiliations:** 1 The Assessment and Intervention Center for Autistic Children, Guangzhou Women and Children’s Medical Center, Guangzhou Medical University, Guangdong Provincial Clinical Research Center for Child Health, Guangzhou, China; 2 Department of Behavioral Development, Guangzhou Women and Children’s Medical Center, Guangzhou Medical University, Guangdong Provincial Clinical Research Center for Child Health, Guangzhou, China

**Keywords:** autism spectrum disorder, nighttime awakenings, sleep characteristics, sleep duration, social jetlag

## Abstract

**Objective:**

This study aims to compare the differences in sleep characteristics between autistic children and typically developing children, with a focus on sleep timing, duration, and patterns.

**Methods:**

This study included 246 children with autism from an autism cohort and 246 age-matched typically developing children selected as controls. Sleep-related data were collected using structured questionnaires and interviews, along with demographic, socioeconomic, and sleep-related variables. Sleep duration was expressed in hours, and sleep timing variables were converted into decimal hours for analysis. Multivariable linear and logistic regression analyses were performed to assess between-group differences after adjusting for relevant confounding factors.

**Results:**

In the weekday sleep pattern, children with autism showed later sleep onset (adjusted *β* = 0.55, 95% CI: 0.27–0.82) and later wake-up time (adjusted *β* = 0.41, 95% CI: 0.25–0.58), along with shorter sleep duration (adjusted *β* = −0.26, 95% CI: −0.47–−0.04). In the weekend sleep pattern, children with autism exhibited significantly later wake-up time (adjusted *β* = 0.55, 95% CI: 0.32–0.79) and shorter sleep duration (adjusted *β* = –0.29, 95%CI:–0.56–−0.03). Regarding circadian sleep timing characteristics, autism status was positively associated with sleep midpoint on both weekdays (adjusted *β* = 0.54, 95% CI: 0.39–0.70) and weekends (adjusted *β* = 0.75, 95% CI: 0.55–0.95) and was also significantly associated with a later corrected mid-sleep point on free days (MSFsc) (adjusted *β* = 0.77, 95% CI: 0.57–0.97).

**Conclusion:**

Children with autism exhibit notable sleep impairments, characterized by later sleep timing, shorter sleep duration, and more frequent nighttime awakenings. These findings suggest that sleep characteristics may be relevant for early identification and management in children with autism.

## Introduction

Autism Spectrum Disorder (ASD) is a neurodevelopmental disorder that typically manifests in early childhood with impairments in social communication, limited behavior patterns, and repetitive behaviors (1). In recent years, the global prevalence of autism has risen significantly (2–6), placing a considerable burden on both public health and the economy (7). Concurrently, there has been an increasing focus on neurodevelopmental comorbidities associated with autism (8), such as sleep disorders (9). This underscores the crucial importance of early diagnosis of these comorbidities to facilitate the implementation of comprehensive and targeted interventions.

Sleep disorders are common comorbidities in individuals with autism (9). Previous studies indicate that the risk of sleep disorders in children with autism is significantly higher, often up to three times greater than in typically developing children (10). These sleep disturbances can significantly affect children's daily functioning, particularly in areas such as attention, learning, memory, and behavior regulation (11). Additionally, sleep issues in children with autism are often accompanied by other physiological and medical conditions (12, 13), further intensifying their overall health challenges.

Although the preschool years are a critical period for early intervention in autism, studies on sleep issues in children with autism during this stage are still limited, and previous studies in this age group typically involve relatively small sample sizes (14, 15). Most existing studies focus on school-age children and adolescents (9, 16), neglecting the unique needs of the preschool period and failing to implement appropriate interventions for comorbid sleep disorders may hinder effective treatment. This study aims to investigate the sleep characteristics and to explore their clinical correlates within the autism-specific cohort, with the goal of providing evidence to inform early identification and intervention strategies.

## Methods

### Study population

Autistic children were recruited between September 2018 and February 2025 by the Behavioral Development Department and Neurology Department of Guangzhou Women and Children's Medical Center, the national regional Children's Medical Center in China. Autistic children were diagnosed by two independent specialist clinicians, based on the following criteria: 1) the Diagnostic and Statistical Manual of Mental Disorders, Fifth Edition (DSM-5) (1). 2) the Autism Diagnostic Observation Schedule (ADOS) (17) and the Autism Diagnostic Interview-Revised (ADI-R) (18), which have been proven to have high reliability and validity in diagnoses of autism in children. 3) The age was less than 6 years. Children initially diagnosed before the age of two years were followed up to obtain a definitive diagnosis when they reached at least two years of age. Age-matched typically developing children were recruited from local kindergartens. Children with neurodevelopmental disorders and other genetic, endocrine, or relevant medical conditions were excluded based on reports from both parents and kindergarten teachers. Written consent was obtained from parents/guardians. The study was approved by the Guangzhou Women and Children's Medical Center Ethics Committee.

### Sleep characteristics

This study utilized a structured parent-report questionnaire based on the validated Chinese version of the Children's Sleep Habits Questionnaire (CSHQ) (19), which was developed from the original CSHQ (20). The CSHQ is a widely used instrument in pediatric sleep research and has been validated in Chinese populations. The questionnaire was administered through face-to-face interviews to ensure data completeness and accuracy. Information on sleep patterns was collected from parents. including: sleep onset time (time of falling asleep) and wake-up time (defined as final morning awakening) on weekdays and weekends, occurrence of nighttime awakenings, frequency of nighttime awakenings (categorized as “rarely [0–1 times/week],” “sometimes [2–4 times/week],” or “often [5–7 times/week]”), as well as a binary classification (“0–1 times/week” vs. “more than once/week”) and the duration of each awakening. All time variables (sleep onset, wake-up time, and sleep midpoint) were originally recorded in hh:mm (hours:minutes) format and converted into decimal hours using the formula: hours + minutes/60. Sleep duration was expressed in hours. Sleep duration was defined as wake-up time minus sleep onset time minus the total duration of nocturnal awakenings. The total duration of nocturnal awakenings was calculated as either the number of awakenings multiplied by the average duration per awakening, or alternatively as the total parent-reported duration of nighttime awakenings. The sleep midpoint was calculated as the midpoint between sleep onset and wake-up time. Social jet lag was defined as the absolute difference between sleep midpoints on weekends and weekdays. Chronotype was represented by the corrected mid-sleep point on free days (MSFsc), which reflects an individual's diurnal preference. When weekday sleep duration was equal to or longer than weekend sleep duration, MSFsc was defined as the weekend sleep midpoint. Otherwise, MSFsc was calculated as the weekend sleep midpoint minus half of the difference between weekend sleep duration and the average weekly sleep duration (21). Sleep characteristics also included the differences between weekends and weekdays in sleep onset and wake-up times.

### Potential confounders

The following characteristics were included as potential confounders. 1) Children's characteristics: child gender(male/female), birth weight (kg), birth length (cm), delivery mode (vaginal/cesarean), gestational age (preterm/full-term/post-term), body mass index (BMI, calculated as weight in kilograms divided by the square of height in meters), and neonatal intracranial hemorrhage (yes/no). Maternal characteristics: maternal age at childbirth, and maternal education level (high school or below, bachelor's degree, or master's degree and above).

### Statistical analysis

Descriptive statistics were employed to characterize sleep patterns in children with autism and typically developing children. Continuous sleep variables were summarized as mean ± standard deviation and compared between groups using independent samples *t*-tests. Categorical sleep variables, such as nighttime waking frequency categories, were reported as proportions and analyzed using chi-square tests.

To examine the relationship between autism and sleep parameters, multivariable linear regression was used for continuous sleep variables, and logistic regression was applied to binary sleep variables. All models were adjusted for child age, sex, gestational age at birth, delivery mode, birth weight, maternal age at delivery, and maternal education level. Potential confounders were identified *a priori* based on existing epidemiological evidence and their known or plausible associations with perinatal and sociodemographic factors as well as neurodevelopmental outcomes. To evaluate the robustness of the main findings and explore potential effect modification by maternal education, we performed stratified sensitivity analyses according to maternal education level. Maternal education was selected because it is an important indicator of socioeconomic status and has been associated with neurodevelopmental outcomes.

A two-sided *p*-value < 0.05 was defined as statistically significant. All analyses were conducted using SAS version 9.4 (SAS Institute Inc., Cary, NC, USA).

## Results

### Baseline characteristics of the study

A total of 246 children with autism were included in this study, with typically developing children individually matched in a 1:1 ratio based on age. Compared with the typically developing group, the autistic cohort had a higher proportion of males (84.17%). In addition, mothers in the autistic group were slightly younger (30.00 ± 4.55 years) and had lower educational attainment. No significant differences were observed between the two groups in other baseline characteristics (Table 1).

**Table 1:** Baseline characteristics of children with autism and typically developing children.

Baseline characteristics	Typically Developing Children	Autistic Children	t/*χ*² value	*P value*
Children's Characteristics				
Age (years), mean ± SD	3.75 ± 1.2	3.56 ± 1.24	1.72	0.087
Sex, *n* (%)			30.84	<0.001
Male	152 (63.6)	208 (85.6)		
Female	87 (36.4)	35 (14.4)		
Body mass index (kg/m2), mean ± SD	16.02 ± 2.63	15.91 ± 2.27	0.46	0.643
Gestational Age at Birth, *n* (%)			1.10	0.578
Preterm (<37 weeks)	22 (9.36)	29 (12.34)		
Term (37–42 weeks)	201 (85.53)	195 (82.98)		
Postterm (>42 weeks)	12 (5.11)	11 (4.68)		
Delivery Mode, *n* (%)			0.37	0.542
Vaginal delivery	157 (64.34)	148 (61.67)		
Cesarean section	87 (35.66)	92 (38.33)		
Birth length (cm), mean ± SD	49.78 ± 3.21	49.85 ± 4.34	−0.2	0.845
Birth weight (kg), mean ± SD	3.14 ± 0.54	3.11 ± 0.57	0.56	0.577
Neonatal Intracranial Hemorrhage, *n* (%)			0.01	0.938
No	227 (92.65)	221 (92.47)		
Yes	18 (7.35)	18 (7.53)		
Maternal Characteristics				
Maternal age (years), mean ± SD	30.89 ± 4.57	30.00 ± 4.55	2.12	0.034
Maternal Education Level, *n* (%)			61.79	<0.001
Junior high school or below	11 (4.49)	73 (30.04)		
High school	24 (9.8)	32 (13.17)		
Bachelor's degree or higher	210 (85.71)	138 (56.79)		

SD, Standard Deviation; Kg, Kilogram; cm, Centimeter; n, Number.

### Sleep characteristics between children with autism and typically developing children

Compared with typically developing children, children with autism spectrum disorder exhibited significant differences across multiple sleep domains, particularly in sleep timing, sleep midpoint, nocturnal awakenings, and circadian sleep-wake regulation. On weekdays, children with autism showed significantly later sleep onset (22:04 ± 2:32 vs. 21:41 ± 0:41, *p* = 0.026) and delayed wake-up times (7:42 ± 0:55 vs. 7:16 ± 0:23, *p* < 0.001). In addition, sleep duration was significantly shorter in the autistic group (9.28 ± 0.84 vs. 9.55 ± 0.64, *p* = 0.005). On weekends, no significant between-group difference was observed in sleep onset; however, children with autism still exhibited significantly later wake-up times (8:19 ± 1:08 vs. 7:44 ± 0:36, *p* < 0.001) and slightly shorter sleep duration (9.40 ± 0.97 vs. 9.74 ± 0.72, *p* = 0.006). Regarding circadian sleep timing, sleep midpoints were significantly delayed in children with autism on both weekdays and weekends (weekday: 3:02 ± 0:50 vs. 2:28 ± 0:24; weekend: 3:37 ± 0:56 vs. 2:51 ± 0:31 both *p* < 0.001). MSFsc was also significantly higher in the autistic children (3.53 ± 0.81 vs. 2.75 ± 0.54, *p* < 0.001). However, no significant differences were observed in social jetlag or weekend–weekday variability measures (*p* > 0.05).

In terms of nocturnal awakenings, autistic children showed a significantly higher frequency of nighttime awakenings (*χ*^2^ = 17.45, *p* < 0.001), with a greater proportion reporting regular awakenings (5–7times/week; 19.82%vs.7.17%). A binary classification similarly indicated a higher prevalence of nighttime awakenings in the autistic group (32.6% vs. 18.14%, *p* < 0.01), whereas no significant difference was found in the duration of individual awakenings (*p* = 0.687) (Table 2).

**Table 2:** Comparison of sleep characteristics between children with autism and typically developing children.

Sleep Characteristics	Typically Developing Children	Autistic Children	*t/χ*^2^ *value*	*P value*
Weekday Sleep Patterns				
Sleep onset (decimal hours), mean ± SD	21.7 ± 0.7	22.08 ± 2.54	−2.23	0.026
Wake-up time (decimal hours), mean ± SD	7.27 ± 0.39	7.72 ± 0.92	−5.97	<0.001
Sleep duration (hours), mean ± SD	9.55 ± 0.64	9.28 ± 0.84	2.83	0.005
Weekend Sleep Patterns				
Sleep onset (decimal hours), mean ± SD	22.05 ± 0.81	22.06 ± 3.43	−0.03	0.978
Wake-up time (decimal hours), mean ± SD	7.75 ± 0.6	8.33 ± 1.14	−5.81	<0.001
Sleep duration (hours), mean ± SD	9.74 ± 0.72	9.40 ± 0.97	2.79	0.006
Sleep Timing Variability				
Weekend–weekday sleep onset difference (hours), mean ± SD	0.36 ± 0.59	0.28 ± 0.78	1.07	0.285
Weekend–weekday wake time difference (hours), mean ± SD	0.48 ± 0.57	0.44 ± 0.70	0.53	0.596
Social jetlag (hours), mean ± SD	0.47 ± 0.37	0.45 ± 0.51	0.37	0.715
Sleep Timing Characteristics				
Weekday sleep mid-point (decimal hours), mean ± SD	2.48 ± 0.4	3.04 ± 0.83	−8.02	<0.001
Weekend sleep mid-point (decimal hours), mean ± SD	2.87 ± 0.53	3.62 ± 0.95	−8.58	<0.001
MSFsc (hours), mean ± SD	2.75 ± 0.54	3.53 ± 0.81	−7.51	<0.001
Night Wakings Patterns				
Awakes during night, *n* (%)			17.45	<0.001
Rarely (0 to 1 time/week)	194 (81.86)	153 (67.4)		
Sometimes (2 to 4 times/week)	26 (10.97)	29 (12.78)		
Usually (5 to 7 times/week)	17 (7.17)	45 (19.82)		
Awakes during night, *n* (%) [Binary Classification]			12.85	<0.001
0 to 1 time/week	194 (81.86)	153 (67.4)		
More than 1 time/week	43 (18.14)	74 (32.6)		
Duration per night waking (hours), mean ± SD	0.38 ± 0.34	0.45 ± 0.44	−0.41	0.687

SD, Standard Deviation; n, Number; MSFsc, Corrected mid-sleep point on free days.

All sleep timing variables (sleep onset, wake-up time, and sleep midpoint) were originally recorded in hh:mm (hours:minutes) format and converted into decimal hours (hours + minutes/60) for analysis. Sleep duration is expressed in hours.

### Association between autism status and sleep characteristics

In the weekday sleep pattern, children with autism showed later sleep onset (adjusted *β* = 0.55, 95% CI: 0.27–0.82) and later wake-up time (adjusted *β* = 0.41, 95% CI: 0.25–0.58), along with shorter sleep duration (adjusted *β* = −0.26, 95% CI: −0.47− −0.04).

In the weekend sleep pattern, children with autism exhibited significantly later wake-up time (adjusted *β* = 0.55, 95% CI: 0.32–0.79) and shorter sleep duration (adjusted *β*= −0.29, 95% CI: −0.56– −0.03).

Regarding circadian-related sleep timing characteristics, autism status was positively associated with a later sleep midpoint on both weekdays (adjusted *β* = 0.54, 95% CI: 0.39–0.70) and weekends (adjusted *β* = 0.75, 95% CI: 0.55–0.95) and as well as with MSFsc (adjusted *β* = 0.77, 95% CI: 0.57–0.97), suggesting later sleep timing. However, no significant associations were observed between autism and social jetlag or nocturnal awakening duration (Table 3).

**Table 3:** Associations of autism with sleep characteristics.

Sleep characteristics	Crude *β* (95% CI)	Adjusted *β* (95% CI)^a^
Weekday Sleep Patterns		
Sleep onset	**0.38** (**0.05–0.72)**	**0.55** (**0.27–0.82)**
Wake-up time	**0.44** (**0.30–0.59)**	**0.41** (**0.25–0.58)**
Sleep duration	**−0.27**(**−0.45– −0.09)**	**−0.26**(**−0.47– −0.04)**
Weekend Sleep Patterns		
Sleep onset	0.01(−0.47–0.48)	0.18(−0.25–0.61)
Wake-up time	**0.58** (**0.38–0.78)**	**0.55** (**0.32–0.79)**
Sleep duration	**−0.34**(**−0.56– −0.12)**	**−0.29**(**−0.56– −0.03)**
Sleep Timing Variability		
Weekend–weekday sleep onset difference	−0.08(−0.22–0.06)	−0.03(−0.20–0.14)
Weekend–weekday wake time difference	−0.04(−0.2–0.11)	−0.08(−0.26–0.09)
Social jetlag	−0.02(−0.14–0.09)	−0.07(−0.20–0.06)
Sleep Timing Characteristics		
Weekday sleep mid-point	**0.56** (**0.42–0.70)**	**0.54** (**0.39–0.70)**
Weekend sleep mid-point	**0.75** (**0.58–0.93)**	**0.75** (**0.55–0.95)**
MSFsc	**0.78** (**0.61–0.95)**	**0.77** (**0.57–0.97)**
Duration per night waking	0.08(−0.31–0.46)	0.15(−0.30–0.60)

a Adjusted for child's age, sex, gestational age at birth, delivery mode, birth weight, maternal age and maternal education level.

All sleep timing variables (sleep onset, wake-up time, and sleep midpoint) were originally recorded in hh:mm (hours:minutes) format and converted into decimal hours (hours + minutes/60) for analysis. Sleep duration is expressed in hours.

CI, Confidence Interval; MSFsc, Corrected mid-sleep point on free days.

Bold indicates statistical significance.

In addition, children with autism had significantly higher odds of nighttime wakings (adjusted OR =  2.31, 95% CI: 1.38–3.87) (Table 4). However, no significant association was observed for the duration of individual nighttime awakenings (Table 4).

**Table 4:** Association between autism and night wakings.

Sleep characteristics	Crude OR (95%CI)	Adjusted OR (95% CI)^a^
Night Wakings		
Typically Developing Children	ref	ref
Autistic children	**2.18 (1.42–3.36)**	**2.31 (1.38–3.87)**

a Adjusted for child's age, sex, gestational age at birth, delivery mode, birth weight, maternal age and maternal education level.

Night waking frequency was binary coded (0∼1 vs. >1 time/week), with >1 time/week as the reference category.

OR, Odds Ratio; CI, Confidence Interval.

Bold indicates statistical significance.

### Sensitivity analysis

Sensitivity analyses using stratification showed that the overall association patterns across maternal education subgroups were largely consistent with the main analysis, with relatively stronger associations observed among children of mothers with lower educational attainment (Supplementary Table S1).

## Discussion

Children with autism and typically developing children showed significant differences in sleep patterns, characterized by later wake-up times and shorter overall sleep duration. These differences were evident on both weekdays and weekends, with children with autism continuing to obtain less sleep even on weekends. In addition, children with autism experienced more frequent nighttime awakenings. Furthermore, circadian-related indicators suggested later sleep timing among children with autism, including a later sleep midpoint and higher MSFsc. Together, these findings suggested that sleep disturbances in preschool children with autism were primarily characterized by later sleep timing and insufficient sleep, accompanied by an increased frequency of nighttime wakings.

Previous studies showed that adolescents with autism often exhibited delayed sleep onset, shorter sleep duration, and frequent nighttime awakenings, which significantly affected their sleep quality (16, 22, 23). Our study found that, compared to typically developing children, preschool-aged children with autism had shorter sleep durations on both weekdays and weekends, consistent with earlier studies. Additionally, the study indicated later sleep timing in preschool-aged children with autism, as reflected by sleep midpoint and MSFsc. Taylor et al (24) also reported that older adolescents with autism typically exhibited a later chronotype. Based on the autism cohort and the concurrent assessment of sleep at the time of autism diagnosis, our study suggested that sleep problems may have emerged alongside the diagnosis and could have manifested as an early comorbid symptom. This underscored the potential role of sleep disturbances as an early accompanying symptom of autism. The sleep problems observed in children with autism may not be isolated behavioral issues, but rather closely tied to the neurodevelopmental characteristics of the disorder itself.

Previous studies suggested that adolescents with autism may exhibit “night owl” chronotypes (24) and an increase in social jetlag with age (25). Our study found that, although no significant social jetlag was observed in preschool-aged children with autism, circadian-related indicators suggested later sleep timing, as reflected by a later sleep midpoint and higher MSFsc, which is consistent with later sleep timing patterns. These findings indicated a tendency toward later sleep timing in overall sleep patterns, which was largely consistent with previous findings (24). No significant social jetlag was observed in preschool-aged children with autism, which was not consistent with findings from previous studies (25). This may be due to weaker school-and society-imposed schedule constraints in preschool children, resulting in minimal weekday–weekend differences and a reduced basis for social jetlag. In addition, circadian rhythm is age-dependent, and social jetlag typically becomes more evident during school age and adolescence as misalignment between social demands and endogenous biological rhythms becomes more pronounced. Thus, social jetlag may not yet be fully established in early developmental stages. Furthermore, the predominantly family-based sleep schedules in our cohort may have further limited weekend compensation effects, thereby attenuating the expression of social jetlag.

The sleep problems of autistic children have been explained by the following mechanisms: First, genetic and biological factors, such as autism-associated genetic variants, may influence melatonin secretion and neurotransmitter systems, potentially contributing to circadian rhythm disruption (26, 27); Second, stereotypical behaviors and rigid routines in children with autism likely made their sleep patterns more fixed, limiting their ability to adapt (28); Third, sensory processing differences may have caused hypersensitivity to external stimuli, such as light and noise, interfering with sleep (10, 29); Last, elevated anxiety levels and difficulties with emotional regulation may have worsened nighttime sleep disturbances (29, 30).

The preschool period is a critical stage for brain development, during which the brain's high plasticity makes timely interventions particularly important. Sleep plays a vital role in neurodevelopment, cognitive function, emotional regulation, and behavior (31, 32). However, children with autism frequently experience significant sleep disturbances, which can worsen the core symptoms of autism (33). Therefore, early intervention to address sleep issues is essential, as it can foster neurodevelopment and support behavioral progress in children with autism. At present, sleep issues are often overlooked in the interventions for autistic children. For preschool-aged children with autism who face sleep challenges, addressing these concerns should be a key focus of early behavioral interventions to enhance their sleep patterns and overall sleep quality.

### Strengths and limitations

This study had several notable strengths. First, it utilized data from an autism-specific cohort, with all autism diagnoses being clinically confirmed using standardized tools, including the DSM-5, the Autism Diagnostic Observation Schedule (ADOS), and the Autism Diagnostic Interview-Revised (ADI-R), with continuous follow-up to ensure both diagnostic validity and accuracy. Second, the study focused on sleep issues in young children with autism. Given the significant differences in brain development and sleep patterns between younger and older children, the findings were crucial for understanding sleep characteristics during the early stages of autism and provided the scientific basis for early intervention. Finally, the study found that sleep symptoms were present in children with autism at the time of diagnosis, suggesting sleep problems may be intrinsically linked to the core symptoms of autism. This finding supported that autism may involve subtypes associated with specific sleep disorders, providing new insights for clinical classification and individualized interventions.

This study had several limitations that should be considered. First, the sample was predominantly composed of Han Chinese children, which limits the generalizability of the findings to other ethnic groups. Second, the data collection period overlapped with the COVID-19 pandemic. Pandemic-related changes in social environment and daily routines (e.g., home-based learning, school closures, and altered family schedules) may have influenced children's sleep patterns. Although both autistic children and typically developing children were recruited during the same period, which may reduce between-group bias to some extent, the potential impact of pandemic-related factors cannot be fully excluded. Third, while the key confounding factors were adjusted for, certain variables such as electronic device use, screen time, parental sleep habits, medication use (e.g., sleep-inducing or psychotropic medications), and clinical severity were not accounted for and might have influenced the results. Fourth, the study's cross-sectional design precludes the ability to track the evolution of sleep patterns in children with autism over time. Future research should adopt longitudinal study to better understand the developmental trajectory of sleep difficulties in this population. Finally, objective sleep assessment methods, such as actigraphy or polysomnography (PSG), were not employed. Incorporating these techniques in future studies could yield more precise and reliable data on sleep characteristics in children with autism.

## Conclusion

This study found significant differences in the sleep patterns of preschool-aged children with autism compared to typically developing children, including later sleep onset, shorter overall sleep durations, and more frequent nighttime awakenings, Additionally, children with autism exhibited later sleep timing. These findings underscore the importance of timely intervention for sleep problems in preschool-aged children with autism.

## Data Availability

The original contributions presented in the study are included in the article/Supplementary Material, further inquiries can be directed to the corresponding author/s.
