# Supplementary material for: Sleep characteristics in young children with autism spectrum disorder: a case control study
Source: Front Pediatr. 2026 Sep 2;14:1742989. doi: 10.3389/fped.2026.1742989 (PMC13579451; doi:10.3389/fped.2026.1742989)
Supplement: Supplementary file 1 [file Table1.docx]

| **Supplementary Table1 Association of Risk Factors for Sleep Problems in Children with Autism Stratified by Maternal Education Level** | | | |  |
| --- | --- | --- | --- | --- |
|  | **Maternal Education Level** | | | **P for  interaction** |
|  | **Junior high school  or below** | **High school** | **Bachelor's degree or higher** |  |
| **Weekday Sleep Patterns** |  |  |  |  |
| Sleep onset | 0.72(0-1.45) | 1.51(0.90-2.12) | 0.37(0.03-0.7) | **0.022** |
| Wake-up time | 0.69(-0.1-1.49) | 1.01(0.33-1.68) | 0.27(0.11-0.44) | 0.016 |
| Sleep duration | 0.41(-0.37-1.19) | -0.72(-1.53-0.08) | -0.19(-0.43-0.05) | **0.584** |
| **Weekend Sleep Patterns** |  |  |  |  |
| Sleep onset | 0.66(-0.21-1.53) | 1.04(0.28-1.79) | -0.06(-0.61-0.48) | 0.207 |
| Wake-up time | 1.29(0.08-2.49) | 0.72(-0.1-1.53) | 0.53(0.28-0.78) | 0.725 |
| Sleep duration | -0.67(-1.77-0.44) | -0.07(-1.24-1.11) | -0.28(-0.57-0.01) | 0.322 |
| **Sleep Timing Variability** |  |  |  |  |
| Weekend–weekday sleep onset difference | -0.27(-0.93-0.4) | -0.34(-0.86-0.17) | 0.04(-0.16-0.24) | 0.53 |
| Weekend–weekday wake time difference | 0.21(-0.72-1.14) | -0.43(-0.74--0.12) | -0.06(-0.26-0.15) | 0.35 |
| Social jetlag | 0.31(-0.2-0.82) | **-0.64(-1.23--0.05)** | -0.09(-0.24-0.06) | 0.121 |
| **Sleep Circadian Phase Patterns** |  |  |  |  |
| Weekday sleep mid-point | 0.72(-0.01-1.45) | 1.27(0.72-1.82) | 0.37(0.21-0.53) | <0.01 |
| Weekend sleep mid-point | 1.21(0.5-1.92) | 1.18(0.34-2.03) | 0.69(0.45-0.93) | 0.43 |
| MSFsc | 1.26(0.64-1.87) | 0.99(0.06-1.93) | 0.71(0.47-0.96) | 0.70 |
| **Duration per night waking** | -0.09(-1.15-0.97) | - | 0.23(-0.69-1.15) | 0.46 |
| ᵇ Adjusted for child’s age, sex, gestational age at birth, delivery Mode, birth weight, maternal age and maternal education level. | | | |  |
